# Conditional Cripto overexpression in satellite cells promotes myogenic commitment and enhances early regeneration

**DOI:** 10.3389/fcell.2015.00031

**Published:** 2015-05-21

**Authors:** Carolina Prezioso, Salvatore Iaconis, Gennaro Andolfi, Lorena Zentilin, Francescopaolo Iavarone, Ombretta Guardiola, Gabriella Minchiotti

**Affiliations:** ^1^Stem Cell Fate Laboratory, Institute of Genetics and Biophysics “A. Buzzati-Traverso,” CNRNaples, Italy; ^2^Molecular Medicine Laboratory, International Centre for Genetic Engineering and BiotechnologyTrieste, Italy

**Keywords:** Cripto, transgenic mice, satellite cells, muscle regeneration, myogenic commitment

## Abstract

Skeletal muscle regeneration mainly depends on satellite cells, a population of resident muscle stem cells. Despite extensive studies, knowledge of the molecular mechanisms underlying the early events associated with satellite cell activation and myogenic commitment in muscle regeneration remains still incomplete. Cripto is a novel regulator of postnatal skeletal muscle regeneration and a promising target for future therapy. Indeed, Cripto is expressed both in myogenic and inflammatory cells in skeletal muscle after acute injury and it is required in the satellite cell compartment to achieve effective muscle regeneration. A critical requirement to further explore the *in vivo* cellular contribution of Cripto in regulating skeletal muscle regeneration is the possibility to overexpress Cripto in its endogenous configuration and in a cell and time-specific manner. Here we report the generation and the functional characterization of a novel mouse model for conditional expression of Cripto, i.e., the *Tg:DsRed^*loxP/loxP*^Cripto-eGFP* mice. Moreover, by using a satellite cell specific *Cre*-driver line we investigated the biological effect of Cripto overexpression *in vivo*, and provided evidence that overexpression of Cripto in the adult satellite cell compartment promotes myogenic commitment and differentiation, and enhances early regeneration in a mouse model of acute injury.

## Introduction

The responses of skeletal muscle tissue following acute or chronic damages are highly complex and coordinated processes, involving many different cell populations that interact each other to promote muscle regeneration, inflammation and angiogenesis, until full regeneration of the tissue and its functional recovery. This process is tightly controlled by signals released by the damaged fibers, which lead to the activation of the quiescent satellite cells, i.e., the myogenic stem cell population that is among the major players responsible for the regeneration of skeletal muscle (Collins et al., 2005). Following an injury, the satellite cells are activated and proliferate as myogenic progenitors that migrate to the damaged site, differentiate and fuse each other to form new myofibers (Hawke and Garry, 2001; Chargé and Rudnicki, 2004). This sequential process is correlated with the finely regulated expression of the Myogenic Regulatory Factors (MRFs). Indeed, quiescent satellite cells express Pax7 and, upon activation, upregulate the myogenic transcription factor MyoD. Upon commitment to differentiation these transient amplifying cells (Pax7^+^/MyoD^+^) downregulate Pax7 and upregulate differentiation genes, as Myogenin and MRF4, while a subset of these cells downregulate MyoD retaining Pax7 expression to replenish the pool of quiescent satellite cells (Zammit et al., 2004; Olguin et al., 2007; Tajbakhsh, 2009). Beside satellite cells, regeneration process is also orchestrated by the crosstalk between heterogeneous cell populations that are recruited/activated after damage, such as the inflammatory cells that are always associated with tissue regeneration, supporting myogenic progression by interacting with satellite cells (Kharraz et al., 2013; Saclier et al., 2013). Besides extensive studies, knowledge of the mechanisms underlying this highly orchestrated process and how the different cell populations establish their fate is still far to be fully elucidated, and much less is known on the extrinsic regulation of this process (Bentzinger et al., 2010). We have recently demonstrated that Cripto, a key regulator of early vertebrate embryogenesis (Shen and Schier, 2000; Minchiotti et al., 2001), is a new player in this process (Guardiola et al., 2012). Cripto is a GPI-anchored protein (Minchiotti et al., 2001) and a developmental factor required for correct orientation of the anterior–posterior axis in the vertebrate embryos (Chu et al., 2005; Minchiotti, 2005). Despite its well-characterized role in embryo development and embryonic stem cell differentiation (Minchiotti, 2005), the role of Cripto in adult life has been poorly investigated also due to the fact that *Cripto* knockout mice are embryonic lethal (Ding et al., 1998; Xu et al., 1999), and that its expression is almost absent in adult physiological conditions. Indeed, Cripto expression is undetectable in skeletal muscles under baseline conditions. However, it becomes rapidly and transiently re-expressed after acute injury, both in myogenic and inflammatory cells, and it is required in the myogenic compartment to achieve an efficient regeneration (Guardiola et al., 2012). Interestingly, a soluble form of the protein (sCripto) is able to rescue the effect of genetic inactivation of *Cripto*, thus recapitulating the function of endogenous membrane form of the protein (Guardiola et al., 2012). Yet, the cellular contribution of Cripto in skeletal muscle repair remains to be further clarified, and its knowledge is limited also by the absence of mouse models for conditional and tissue–specific Cripto expression. Here we report the generation and characterization of novel transgenic mice for conditional expression of *Cripto* in its endogenous configuration, which allowed us to study the biological effect of satellite cell-specific *Cripto* overexpression on muscle regeneration and myogenic cell fate determination.

## Results

### Generation of conditional cripto gain of function transgenic mice

**Figure 1:**
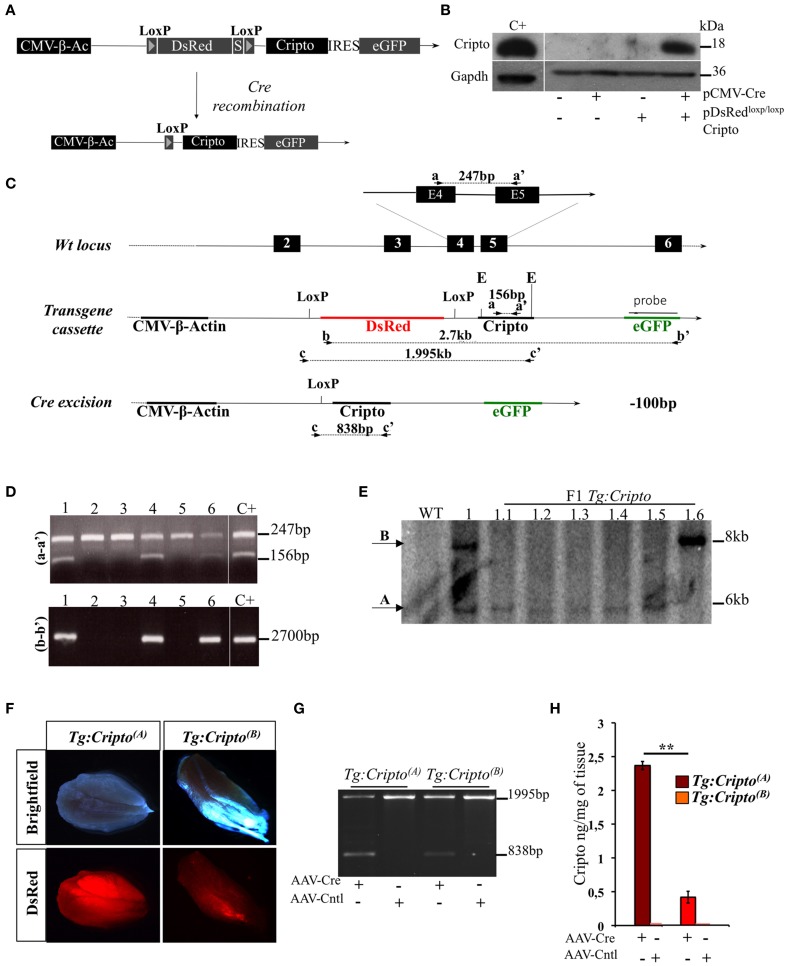
**(A)** Schematic representation of the pDsRed^*loxP/loxP*^Cripto targeting vector. The nuclear DsRed gene flanked by two loxP sites and followed by three termination sequences (S) is transcribed by a constitutive CMV-β-Actin (CMV-β-Ac) promoter. Upon *cre*- mediated recombination, the DsRed sequence is excised and the Cripto-IRES-eGFP cassette is transcribed from the CMV/β-actin promoter. **(B)** Western blot analysis using anti-Cripto antibody on total protein extracts of HEK-293T cells transfected with pDsRed^*loxP/loxP*^Cripto vector either alone or in combination with pCMV-Cre vector. Gapdh was used as a loading control. Protein extracts from undifferentiated mouse embryonic stem cells (ESCs) were used as positive control (C+). **(C)** Schematic representation of the genotyping strategy. Location of PCR primers (black arrowheads) and the probe (black bar) used for genotyping are indicated. Numbered boxes represent wild-type exons (black). E, EcoRI. **(D)** Representative PCR screening on tail genomic DNA from mice generated after oocyte injection. Primers a-a′ (top panel) amplified a 247-bp fragment of the wild-type *Cripto* locus and a 156-bp fragment of the *Cripto* transgene. Primers b-b' (bottom panel) amplified a 2700-bp fragment spanning the DsRed-IRES-Cripto cassette. **(E)** Genotyping by Southern blot analysis. Genomic DNA from wild type (WT), founder (1) and F1 offspring mice (from 1.1 to 1.6) was digested with EcoRI and hybridized with eGFP probe shown in **(C)**. The sizes of hybridized fragments are indicated in kilobases. **(F)** Representative pictures of direct fluorescence of freshly isolated skeletal muscle from *Tg:Cripto^(A)^* and *Tg:Cripto^(B)^* mice, showing different levels of DsRed expression. **(G)** PCR screening of biopsy from *Tg:Cripto^(A)^* and *Tg:Cripto^(B)^* TA muscles infected with either AAV-Cre or AAV-Control (AAV-Cntl). Primers c-c' **(C)** amplified a 1995-bp fragment of the transgenic allele and a 838-bp fragment of the recombining allele. **(H)** ELISA assay of Cripto protein levels expressed as ng/mg of tissue in *Tg:Cripto^(A)^* and *Tg:Cripto^(B)^* muscles infected with AAV-Cre and AAV-Cntl (2.36 ± 0.06 ng/mg in *Tg:Cripto^(A)^* vs. 0.43 ± 0.09 ng/mg in *Tg:Cripto^(B)^*; ^**^P ≤ 0.005).

To get insight into the cellular contribution of Cripto in skeletal muscle regeneration, and to finely modulate Cripto expression *in vivo*, we generated a novel transgenic mouse line for conditional *Cripto* expression based on the *Cre*-*loxP* strategy. To generate the pDsRed^*loxP/loxP*^*Cripto* targeting vector, a *Cripto*-IRES-eGFP cassette was inserted downstream of the *DsRed* gene sequence followed by three termination sequences, and flanked by two *LoxP* sites (see Materials and Methods for details; Figure 1A). The effectiveness of the pDsRed^*loxP/loxP*^*Cripto* vector was first evaluated *in vitro*. To this end, HEK-293T cells were transfected with the pCMV-Cre, expressing the Cre recombinase, and the pDsRed^*loxP/loxP*^*Cripto* plasmids, either alone or in combination, and Cripto protein expression was evaluated. We first verified that eGFP expression was induced in cells cotransfected with pDsRed^*loxP/loxP*^*Cripto* and pCMV-Cre (Figure S1A). Accordingly, Cripto protein was specifically induced (Figure 1B) and, as expected, it localized at the cell membrane (Minchiotti et al., 2000) of eGFP expressing cells (Figure S1B). Following the *in vitro* validation of the targeting vector, transgenic mice were generated by pronuclear injection, and the presence of the transgene in the offspring was assessed by PCR genotyping of tail biopsies (Figures 1C,D). One out of three transgenic mice obtained gave germline transmission and carried two copies of the transgene that segregated independently in the offspring (Figure 1E). Two founder lines were thus established and bred to FVB/N *wild type* mice to generate the *Tg:DsRed^*loxP/loxP*^Cripto-eGFP^(A)^* and *Tg:DsRed^*loxP/loxP*^Cripto-eGFP^(B)^* colonies (from now onwards named *Tg:Cripto^(A)^* and *Tg:Cripto^(B)^*, respectively).

### Functional characterization of the Tg:Cripto transgenic lines

Different studies have shown that significative differences exist in the expression level of the same transgene between individual founder siblings, due to different integration loci, and the influence of the genomic sequences flanking the integration site (Palmiter et al., 1982; Overbeek et al., 1986). To characterize *Tg:Cripto^(A)^* and *Tg:Cripto^(B)^* transgenic lines, we first assessed DsRed expression in freshly isolated muscles by direct fluorescence and found a stronger DsRed signal in *Tg:Cripto^(A)^* compared to *Tg: Cripto^(B)^* muscles (Figure 1F). We thus evaluated whether Cripto was expressed upon *Cre*-mediated recombination *in vivo*. To this end, TA muscles of both transgenic lines were injected with Adeno Associated Virus 9 (Inagaki et al., 2006) encoding Cre recombinase (AAV-Cre) or empty vector (AAV-Cntl) as control. Ten days after infection, TA muscles were explanted and genotyped by PCR. As expected, a 838-bp band characterizing the recombining allele was specifically detected in the AAV-Cre injected muscles (Figure 1G), and Cripto protein was induced at different levels as determined by ELISA-based assay (2.36 ± 0.06 ng/mg in *Tg:Cripto^(A)^* vs. 0.43 ± 0.09 ng/mg in *Tg:Cripto^(B)^*; ^**^P = 0.005) (Figure 1H).

All together these data demonstrate that Cripto expression is regulated upon *Cre*-mediated recombination and is induced at different levels in the two transgenic lines.

### Time -dependent effect of cripto overexpression in adult satellite cells on skeletal muscle regeneration

**Figure 2:**
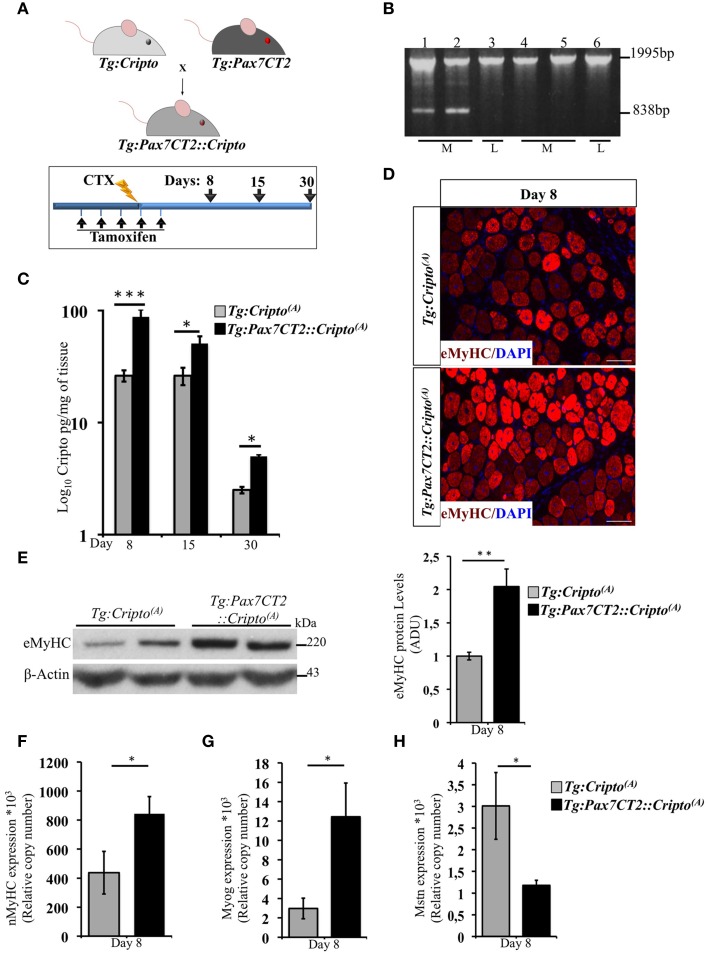
**(A)** Schematic representation of mouse breeding (upper panel) and *Cripto* conditional gain of function strategy in adult satellite cells (lower panel). Tamoxifen were injected i.p., in adult (5 weeks old) *Tg:Pax7CT2::Cripto* mice and *Tg:Cripto* control littermates once a day for 5 days. At day 4, regeneration was triggered by CTX injection in TA muscle of both groups, and analysis performed at the indicated time points (days 8, 15, and 30). The consecutive bars indicate the days. **(B)** PCR screening on muscle genomic DNA showing tamoxifen-induced recombination only in the muscle of *Tg:Pax7CT2::Cripto^(A)^*. Primers c-c′ (See Figure 1C) amplified a 1995-bp fragment of the transgenic allele and a 838-bp fragment of the recombining allele. The recombining allele is detected in the muscle (M; lanes 1, 2) but not in the liver (L; lane 3) of *Tg:Pax7CT2::Cripto^(A)^* mice, and is not detected in the muscle (M; lanes 4, 5) and in the liver (L; lane 6) of *Tg:Cripto^(A)^* control littermates. **(C)** ELISA assay of Cripto protein levels in TA muscles at different time points after CTX injection expressed as pg/mg of muscle tissue at the indicated time points. Values are mean ± SEM of *n* = 5 mice/group. ^*^P ≤ 0.05; ^***^P = 0.0005. **(D)** Representative pictures of embryonic Myosin Heavy Chain (eMyHC) Immunofluorescence on TA sections from *Tg:Pax7CT2::Cripto^(A)^* mice and *Tg:Cripto^(A)^* control littermates at day 8. Scale bars = 100 μm. **(E)** Representative Western blot using anti-eMyHC antibody on total protein extracts of TA muscles from *Tg:Pax7CT2::Cripto^(A)^* mice and their *Tg:Cripto^(A)^* control littermates at day 8 after CTX. β-Actin was used as a loading control. The densitometric analysis is expressed as Arbitrary Densitometric Unit (ADU) expressing the eMyHC/β-Actin ratio. Values are mean ± SEM of *n* = 3 mice/group. ^**^P = 0.005. **(F–H)** qRT-PCR analysis of neonatal Myosin Heavy Chain (nMyHC) **(F)** Myogenin (Myog) **(G)** and Myostatin (Mstn) **(H)** on TA muscle total RNAs from *Tg:Pax7CT2::Cripto^(A)^* mice and the *Tg:Cripto^(A)^* control littermates at day 8 after CTX injection. Values are mean ± SEM, 5 mice/group. ^*^P ≤ 0.05.

**Figure 3:**
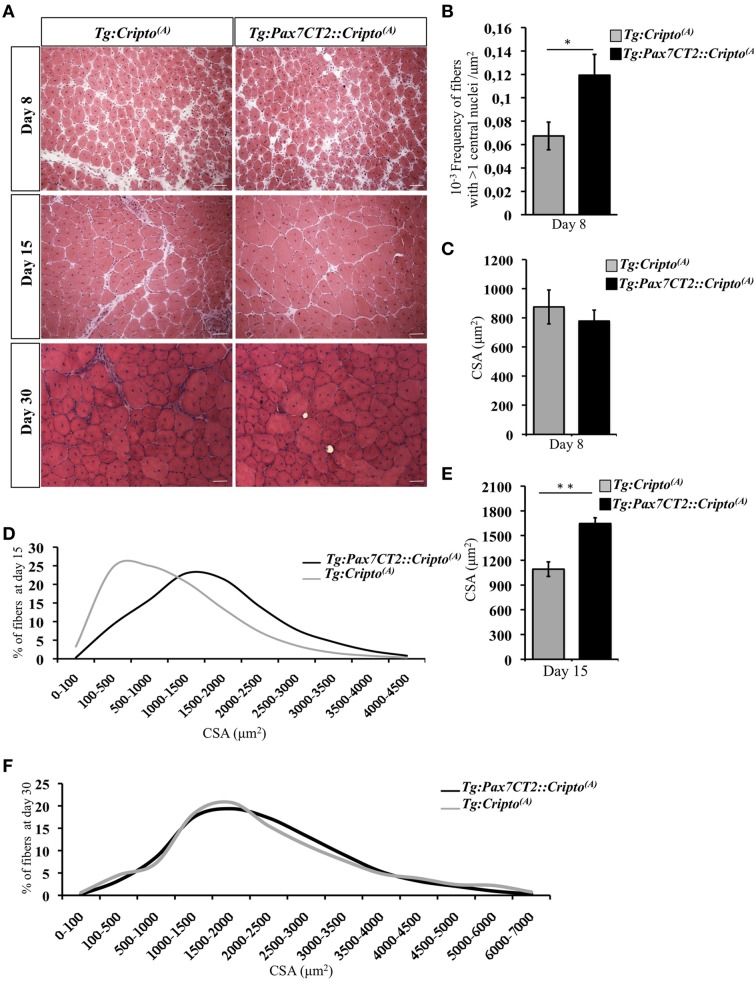
**(A)** Representative H&E staining of TA muscle sections from *Tg:Pax7CT2::Cripto*^(A)^ and *Tg:Cripto^(A)^* TA muscles at the indicated time points. Scale bars = 50μm. **(B)** Morphological analysis on muscle sections at day 8 after injury showing the number of myofibers containing more than one central nuclei/area in each group. Values are mean ± SEM, 5 mice/group ^*^P ≤ 0.05. **(C)** Average of centrally nucleated myofibers size values in TA muscle sections at day 8 after injury. Values are mean ± SEM, 5 mice/group. **(D)** Myofiber Cross Sectional Area distribution at 15 days after CTX injection; 5 mice/group. **(E)** Average of centrally nucleated myofibers size values in TA muscle sections at day 15 after injury of *Tg:Pax7CT2::Cripto*^(A)^ mice and *Tg:Cripto^(A)^* control littermates. Values are mean ± SEM, 5 mice/group. ^**^P ≤ 0.005. **(F)** Myofiber Cross Sectional Area distribution at 30 days after CTX injection; 4 mice/group.

We have recently shown that adenovirus–mediated soluble Cripto (sCripto) overexpression accelerates muscle regeneration induced by acute injury (Guardiola et al., 2012). Nevertheless, viral mediated Cripto expression does not allow us to discriminate between the inflammatory and satellite cell contribution of Cripto overexpression. To overcome this limitation, we crossed *Tg:Cripto^(A)^* and *Tg:Cripto^(B)^* mice with the tamoxifen-inducible *Tg:Pax7CreER^T2^* mice (Mourikis et al., 2012) and obtained the *Tg:Pax7CreER^T2^::DsRed^*loxP/loxP*^Cripto-eGFP^(A)^* and *Tg:Pax7CreER^T2^::DsRed^*loxP/loxP*^Cripto-eGFP^(B)^* trangenic lines (from now onwards named *Tg:Pax7CT2::Cripto^(A)^* and *Tg:Pax7CT2::Cripto^(B)^*) (Figure 2A). We first assessed the effects of satellite cell-specific Cripto overexpression on skeletal muscle regeneration. To this end, *Tg:Pax7CT2::Cripto^(A)^* and *Tg:Pax7CT2::Cripto^(B)^* adult mice and their control littermates were treated with tamoxifen once a day for 5 days; at day 4, muscle regeneration was triggered in TA muscles by local injection of cardiotoxin (CTX; Figure 2A). Genetic recombination was first confirmed by PCR analysis on TA muscle genomic DNA (Figure 2B, Figure S2A), and Cripto protein levels were quantified by ELISA assay on total protein extracts at different time points after injury. Increased Cripto protein levels were detected in both *Tg:Pax7CT2::Cripto^(A)^* and *Tg:Pax7CT2::Cripto^(B)^* mice compared to control, with *Tg:Pax7CT2::Cripto^(A)^* showing the highest levels of Cripto upon *Cre*-mediated recombination (Figure 2C, Figure S2B). To assess if Cripto overexpression might influence muscle regeneration, we first evaluated the expression of the embryonic Myosin Heavy Chain (eMyHC), which is a marker of newly regenerated fibers, at day 8 after injury. Both immunofluorescence and Western blot analysis clearly showed increased eMyHC protein levels in *Tg:Pax7CT2::Cripto^(A)^* mice compared to their control littermates (Figures 2D,E). In line with these findings, expression of both neonatal Myosin Heavy Chain (nMyHC) and the early muscle differentiation marker Myogenin (Myog) similarly increased in the overexpressing mice (Figures 2F,G). Furthermore, expression of Myostatin (Mstn), which is a negative regulator of muscle growth (Thomas et al., 2000), was significantly reduced in *Tg:Pax7CT2::Cripto^(A)^*mice compared to control (Figure 2H). Of note, similar results were obtained from the analysis of the other transgenic line (*Tg:Pax7CT2::Cripto^(B)^*; Figures S2C–F). Morphological analysis of H&E-stained TA muscle sections at day 8 showed that the number of myofibers containing more than one nucleus (*n* >1) significantly increased in Cripto overexpressing mice compared to control (0.12 ± 0.02 for *Tg:Pax7CT2::Cripto^(A)^* vs. 0.06 ± 0.01 for *Tg:Cripto^(A)^* of fibers; ^*^P ≤ 0.05; Figures 3A,B). Interestingly, while there was no significant difference in Cross Sectional Area (CSA) between the two groups at day 8 (Figures 3A,C), later on (i.e., at day 15) both CSA distribution and the relative average values significantly increased in the *Tg:Pax7CT2::Cripto^(A)^* mice compared to control (Figures 3A,D,E). To assess if the positive effect of Cripto overexpression on muscle regeneration was either transient or persistent, we extended the analysis to a later time point (i.e., at day 30). Morphometric analysis of the CSA showed no difference in the distribution of muscle fibers in the two groups at day 30 (Figures 3A,F); thus providing evidence of a time-dependent effect of satellite cell -Cripto overexpression on skeletal muscle regeneration. Most remarkably, similar experiments carried on the *Tg:Pax7CT2::Cripto^(B)^* mice gave comparable results (Figures S3A–F), thus ruling out the possibility of a position-effect of the transgene.

### Conditional overexpression of cripto in satellite cells enhances myogenic differentiation

**Figure 4:**
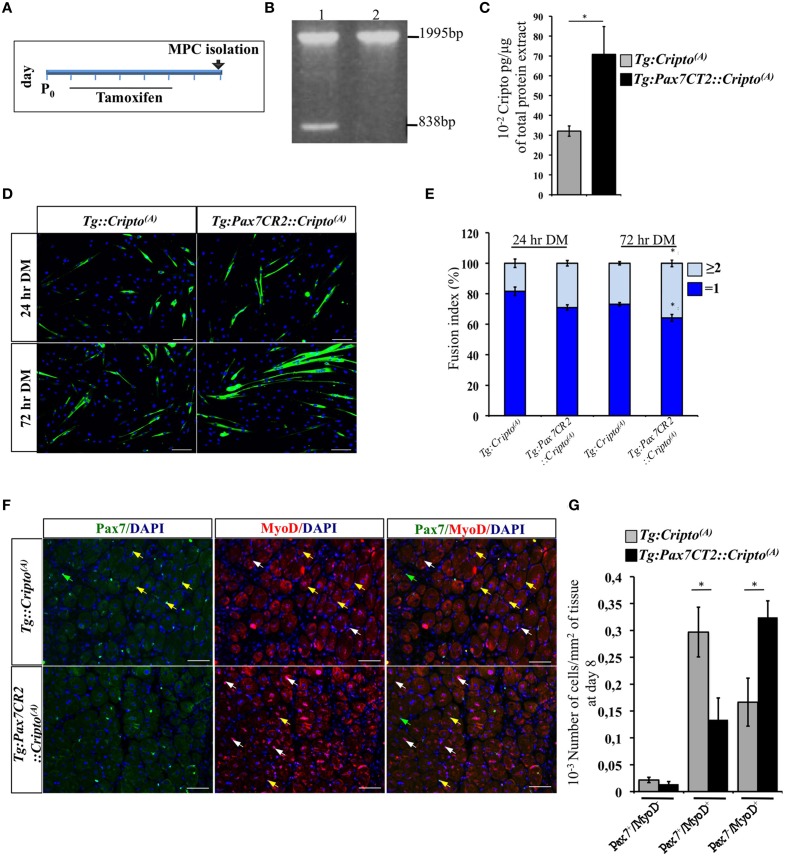
**(A)** Schematic representation of experimental strategy for myogenic progenitor cells (MPCs) isolation. Tamoxifen was injected once a day for 5 days (from P1 to P5) in new-born *Tg:Pax7CT2::Cripto*^(A)^ mice and their control littermates, and MPCs were isolated at P7. **(B)** PCR analysis on muscle genomic DNA showing tamoxifen-induced recombination in *Tg:Pax7CT2::Cripto^(A)^* mice. Primers c-c′ (Figure 1C) amplified a 1995-bp fragment of the transgenic allele and a 838-bp fragment of the recombining allele. **(C)** ELISA assay of Cripto protein levels in primary myoblasts from *Tg:Pax7CT2::Cripto*^(A)^ and control mice expressed as pg/μg of total protein extracts. Values are mean ± SEM of *n* = 3. ^*^P ≤ 0.05. **(D)** Representative pictures of *Tg:Pax7CT2::Cripto^(A)^* and *Tg:Cripto^(A)^* myotubes after 24 and 72 h in differentiation medium (DM) and stained with MF20 antibody. Scale bars = 100 μm. **(E)** Fusion index was calculated as the percentage of MF20^+^ cells with *n* = 2 nuclei at 24 and 72 h in DM. The analysis was performed on 20 independent and random chosen microscope fields. Values are mean ± SEM of 3 independent experiments. ^*^P = 0.05. **(F)** Representative pictures of double immunofluorescence with antibodies anti-Pax7 (green) and anti-MyoD (red) in TA muscles at day 8 after injury. Nuclei were counterstained with DAPI. Arrowheads indicate Pax7^+^/MyoD^−^ (green), Pax7^+^/MyoD^+^ (yellow) and Pax7^−^/MyoD^+^ (white) cells. Scale bars = 100 μm. **(G)** Effect of satellite cells-Cripto overexpression on Pax7^±^/MyoD^±^ cell distribution *in vivo*. The number Pax7^±^/MyoD^±^/area from *Tg:Pax7CT2::Cripto^(A)^* mice and the *Tg:Cripto^(A)^* control littermates was calculated in TA muscles and reported in the chart. Values are mean ± SEM, 5 mice/group. ^*^P ≤ 0.05.

To determine how Cripto overexpression in the satellite cells impact on skeletal muscle regeneration, we first evaluated its effect on early stages of myoblast differentiation *in vitro*. To address this issue, we determined the fusion index (i.e., the percentage of the MF20^+^ cells with *n* ≥ 2 nuclei) of the postnatal myogenic progenitor cells (MPCs) isolated from hindlimb muscles of new-born *Tg:Pax7CT2::Cripto^(A)^* mice and their *Tg:Cripto^(A)^* control littermates at postnatal (P) day 7. *Cre*-mediated recombination was induced by tamoxifen injection (Figure 4A) and assessed by PCR genotyping of muscle tissues (Figure 4B). We then verified that Cripto protein was induced in *Tg:Pax7CT2::Cripto^(A)^* by ELISA assay on postnatal MPCs (70.8 ± 14 ^*^ 10^−2^ pg/μg for *Tg:Pax7CT2::Cripto^(A)^* vs. 32.1 ± 2.6 ^*^ 10^−2^ pg/μg for *Tg:Cripto^(A)^*; ^*^P ≤ 0.05; Figure 4C). Cells were maintained in culture for 24 h in proliferation medium and then shifted to differentiation medium (DM) for 24 and 72 h (Figure 4D). Quantitative analysis showed that the percentage of MF20^+^ cells with *n* ≥ 2 nuclei progressively increased in cultures from *Tg:Pax7CT2::Cripto^(A)^* mice compared to control, at both time points (29.03 ± 1.79% for *Tg:Pax7CT2::Cripto^(A)^* vs. 18.4 ± 2.75% for *Tg:Cripto^(A)^* at 24 h; 35.85 ± 2.24% for *Tg:Pax7CT2::Cripto^(A)^* vs. 26.87 ± 1.08% for *Tg:Cripto^(A)^* at 72 h; ^*^P ≤ 0.05; Figure 4E), thus suggesting that *Cripto* overexpression enhanced *in vitro* myoblasts to myotubes differentiation. In line with these findings, the percentage of Pax^−^/MyoD^+^ cells significantly increased in *Tg:Pax7CT2::Cripto^(A)^* mice compared to control (9.92 ± 1.88% Pax^−^/MyoD^+^ for *Tg:Pax7CT2::Cripto^(A)^* vs. 3 ± 1.62% Pax^−^/MyoD^+^ for *Tg:Cripto^(A)^*; ^*^P ≤ 0.05; Figures S4A,B). Interestingly, these results are in line with data previously reported on isolated myofibers in culture in which addition of sCripto increases the number of Pax7^−^/MyoD^+^ myogenic cell population, promoting satellite cell progression into the myogenic lineage (Guardiola et al., 2012). We thus went on and extended the analysis *in vivo* to assess the effects of Cripto overexpression on the distribution of the satellite cell population during muscle regeneration. To this end, TA muscle sections of *Tg:Pax7CT2::Cripto^(A)^* mice and their control littermates at day 8 after injury (see Figure 2A) were double stained with anti-Pax7 and anti-MyoD antibodies (Figure 4F) and the frequency of (i) quiescent satellite cells (Pax7^+^/MyoD^−^), (ii) transient amplifying cells (Pax7^+^/MyoD^+^), and (iii) cells committed to myogenic differentiation (Pax7^−^/MyoD^+^) was evaluated. Remarkably, the frequency of Pax7^−^/MyoD^+^ cells significantly increased in *Tg:Pax7CT2::Cripto^(A)^* mice compared to control (0.32 ± 0.03 for *Tg:Pax7CT2::Cripto^(A)^* vs. 0.16 ± 0.04 for control of Pax7^−^/MyoD^+^ cells; ^*^P ≤ 0.05; Figures 4F,G), at the expense of Pax7^+^/MyoD^+^, which conversely decreased in *Tg:Pax7CT2::Cripto^(A)^* mice (0.13 ± 0.04 for *Tg:Pax7CT2::Cripto^(A)^* vs. 0.3 ± 0.05 for control of Pax7^+^/MyoD^+^ cells; ^*^P ≤ 0.05; Figures 4F,G); thus providing *in vivo* evidence that adult satellite cell specific Cripto overexpression accelerated myogenic lineage progression.

Taken together our results provide the first *in vivo* evidence that Cripto overexpression in myogenic compartment accelerates the early stages of regeneration process promoting satellite cell myogenic commitment.

## Discussion

Growing evidence link adult tissue repair to reactivation of developmental gene program; nevertheless, our knowledge on the role of key regulators of early vertebrate embryogenesis in tissue regeneration is still limited also by the lack of adequate *in vivo* genetic tools. Indeed, transcription levels and timing of expression of key developmental genes are finely tuned, and experimental strategies to generate constitutive gain-of-function and loss-of-function mouse mutants often result in embryonic lethality. In this scenario, we have focused our attention on the developmental gene Cripto, which is a key regulator of vertebrate embryogenesis. Here we report the generation and functional characterization of a conditional *Cripto* gain-of-function mouse model *Tg:DsRed^*loxP/loxP*^Cripto-eGFP* as a complementary strategy to investigate the effects of Cripto in postnatal and adult life, and potentially in all cell types. Specifically, by crossing *Tg:DsRed^*loxP/loxP*^Cripto-eGFP* mice with a *Tg:Pax7CreER^T2^* mouse line, we have investigated the effect of satellite cell- Cripto overexpression on skeletal muscle regeneration and overcome the limitation of the viral-mediated approach (Guardiola et al., 2012). Consistent with our previous data on isolated myofibers in culture, we provide *in vivo* evidence that satellite cell overexpression of membrane Cripto promotes myogenic commitment and differentiation. However, unlike the viral-mediated overexpression of soluble Cripto (sCripto; Guardiola et al., 2012), the positive effect of satellite cell Cripto overexpression on skeletal muscle regeneration is time-dependent, being restricted to the early phases of regeneration. It is unlikely that the discrepancy in the two approaches is due to protein configuration, i.e., membrane vs. soluble Cripto; indeed, sCripto is able to rescue muscle regeneration defects caused by the genetic ablation of *Cripto* in the satellite cells, indicating that it can recapitulate the role of the endogenous protein (Guardiola et al., 2012). Yet, it is important to consider that Cripto is expressed both in myogenic and inflammatory cells during regeneration. Thus, the different timing of regeneration in the two models might be explained by the fact that while the genetic overexpression is restricted to the satellite cells, the viral-mediated sCripto overexpression has a broader effect and likely affects both the myogenic and the inflammatory cells. Further studies will be necessary to test this hypothesis by deeply investigating the specific contribution of Cripto in inflammatory cells, as well as the autocrine and/or paracrine effects of the endogenous protein.

The positive effect of Cripto overexpression on skeletal muscle regeneration indicated by the increased CSA at day 15 is the expected consequence of its earliest effect on the satellite cell compartment. First, the number of centrally located nuclei is higher in Cripto overexpressing myofibers at day 8 after injury; thus suggesting accelerated differentiation. Accordingly, the myoblast fusion index is increased in postnatal MPCs isolated from Cripto overexpressing mice. Finally, an in depth analysis of Pax7^±^/MyoD^±^ cell distribution shows that the number of Pax7^−^/MyoD^+^ cells committed to differentiation increases both *in vivo* and *in vitro* upon Cripto overexpression. We thus conclude that not only the addition of sCripto protein but also its overexpression in a more physiological context, promotes/accelerates satellite cells commitment to differentiation both *in vitro* and *in vivo*. Taken together our data provide evidence that a timely overexpression of Cripto in the satellite cells promotes myogenic commitment and fusion/differentiation and transiently accelerates regeneration after acute injury. It will be interesting to investigate in future studies the effect of sustained Cripto overexpression on satellite cell self renewal and regeneration after repeated muscle injury.

Finally, in line with the idea that early regeneration is enhanced upon Cripto overexpression, we found that the expression of Myostatin, a member of the TGFβ superfamily which inhibits myoblasts differentiation and acts as a negative regulator of skeletal muscle growth (Amthor et al., 2002; Langley et al., 2002) is downregulated in Cripto overexpressing mice. Interestingly, this Cripto/Myostatin inverse correlation is consistent with previous findings on isolated myofibers in culture showing that sCripto antagonizes Myostatin leading to increased number of myoblast population committed to differentiation (Pax7^−^/MyoD^+^) (Guardiola et al., 2012).

In conclusion, all together these findings broaden our knowledge on the role of Cripto in skeletal muscle regeneration, and add novel insights into its activity in the satellite cell compartment. We believe that these novel mouse models, which also offer the unique opportunity to study the effect of Cripto in the regulation of satellite cell quiescence, activation and self-renewal *in vivo*, will be instrumental to get fundamental insights into the role of Cripto in skeletal muscle homeostasis and regeneration, as an important step toward the therapeutic use of this molecule.

## Materials and methods

### Mice and genotyping

To generate the pDsRed^*loxP/loxP*^Cripto targeting vector, a mouse *Cripto* cDNA (750 bp) (Minchiotti et al., 2001) -IRES-eGFP cassette was cloned downstream of the *Discosoma* sp. red fluorescent protein (DsRed) gene into the pGapDsRed^*loxP/loxP*^ vector, containing the CMV enhancer/chicken beta-actin promoter (CMV/β-Actin) and two loxP sites flanking the DsRed gene upstream of triple STOP-polyA sequences (kindly provided by Dr. Silvia Brunelli). Briefly, a Xho-AflII 2.2 kb DNA fragment spanning the Cripto-IRES-eGFP cassette was excised from the pCripto-IRES-eGFP plasmid (kindly provided by Dr. Giovanna L. Liguori), blunt ended and cloned into the backbone of the pGapDsRed^*loxP/loxP*^ vector previously linearized with EcoR1 and filled in with Klenow Fragment of DNA polymerase I (New England Biolabs). Proper orientation of the inserted Cripto-IRES-eGFP DNA fragment was verified by restriction digestion and DNA sequencing. To generate the transgenic mice, a SalI DNA fragment spanning the entire CMV/β-Actin/DsRed^*loxP/loxP*^CriptoIRES-eGFP transcription unit released from the pDsRed^*loxP/loxP*^Cripto targeting vector was gel purified and injected into fertilized oocytes of FVB/N mice, in collaboration with the Core Facility of Conditional Mutagenesis at San Raffaele Hospital, Milan, Italy. The resulting mice were genotyped by PCR-based screening using primers (a-a′) spanning the *Cripto* gene exon4-exon5 junction for the amplification of both *Cripto* wt (247 bp) and transgenic (156 bp) alleles. The integrity of the transgene was further evaluated by PCR using primers (b-b′) annealing on the DsRed and eGFP sequences (2700 bp). Three out of sixty mice analyzed (5%) were positive for the transgene and only one of them (mouse 1) gave germline transmission of the transgene. Two copies of the transgene, which segregate independently, were identified in the genome of the founder mouse by Southern blot analysis. After germline transmission, heterozygous *Tg:DsRed^*loxP/loxP*^Cripto-eGFP^(A)^* and *Tg:DsRed^*loxP/loxP*^Cripto-eGFP^(B)^* mice colonies were maintained by crossing with wild-type FVB mice and named *Tg:Cripto^(A)^* and *Tg:Cripto^(B)^*, respectively.

For Southern Blot analysis, 20 ug of genomic DNA was prepared from tail biopsies, digested with EcoRI and blotted on Immobilion-Ny+ (Millipore). The ^32^P-labeled probe was a PCR amplified DNA fragment spanning the eGFP insert (546 bp) from the pDsRed^*loxP/loxP*^Cripto vector. The heterozygous *Tg:Pax7CreER^T2^::DsRed^*loxP/loxP*^Cripto-eGFP^(A)/(B)^* mice were generated by crossing *Tg:Pax7CreER^T2^* (Mourikis et al., 2012) with *Tg:Cripto^(A)/(B)^* animals; the offsprings of these crosses were genotyped by double PCR analysis to identify the *Tg:Cripto* (213 bp) and the *Tg:Pax7CreER^T2^* (600 bp) alleles (DsRedCripto for/rev and Pax7Cre for/rev primers). The DNA was prepared from tail biopsy samples of breeding animals. To induce genetic recombination, *Tg:Pax7CreER^T2^::Cripto* and *Tg:Cripto* adult mice (5 weeks old) were injected intraperitoneally (i.p.) with tamoxifen (60 μg/g of body weight per day, Sigma–Aldrich) once a day for 5 days. The genetic recombination was verified by PCR analysis (primers c-c′) to distinguish the transgenic (1995 bp) from the recombining (838 bp) allele. The DNA was obtained from tibialis anterior (TA) muscle biopsy samples. The primer sequences for PCR screening, genotyping and genetic recombination analysis are reported in Table S1.

### Cell culture and transfection

HEK-293T cells were grown in Dulbecco Modified Eagle Medium (DMEM, Gibco) supplemented with 10% Fetal Bovine Serum (FBS, Euroclone) and 1% penicillin-streptomycin (EuroClone), and plated on six-well plates at a density of 2 × 10^5^ cells/cm^2^. Twenty-four hours after plating, cells were transfected with 4 μg of DNA (pDsRed^*loxP/loxP*^Cripto ± pCMV-Cre recombinase expression vector), using Lypofectamin Transfecion Reagent (Life Technologies) following manufacturer's instructions. Forty-eight hours after transfection, cells were fixed for immunofluorescence analysis or visualized by fluorescence microscopy (DMI600; Leica), and images were acquired on an Orca R2 camera (Hamamatsu).

### Muscle injections, preparation, and analysis

Tibialis Anterior (TA) muscles of adult *Tg:Cripto^(A)/(B)^* transgenic mice were injected with either *Cre recombinase*-encoding Adeno Associated Virus (AAV-Cre) or the empty vector (AAV-Cntl) at 1 × 10^12^ genome copies/ml in 10 μl. Muscles were collected after 10 days and total protein extracts were prepared for further analysis. The AAV vectors were prepared by the AAV Vector Unit at ICGEB Trieste (http://icgeb.org/avu-core-facility.html) by packaging AAV2 vector genome backbone into AAV capsid serotype 9, as previously described (Arsic et al., 2003). To induce muscle damage, 10 μl of cardiotoxin (CTX, 70 μM in PBS, Sigma-Aldrich) were injected in the TA muscles of adult (5 weeks old) *Tg:Pax7CreER^T2^::Cripto* and *Tg:Cripto* mice. For morphometric analysis, muscles were harvested at the indicated time points after damage, and frozen in isopentane-cooled liquid nitrogen for cryosection. Myofiber Cross Sectional Area (CSA) was analyzed on haematoxylin/eosin (H&E) stained muscle sections using ImageJ software (freely available software developed at the National Institutes of Health, Bethesda, Maryland, USA). The number of myofibers having more than one nucleus on total area was evaluated, as previously described (Mittal et al., 2010).

All experiments were conducted in strict accordance with the institutional guidelines for animal research and approved by the Department of Public Health, Animal Health, Nutrition and Food Safety of the Italian Ministry of Health in accordance with the law on animal experimentation.

### Western blot

Western blot analysis was performed as previously described (Parisi et al., 2003) on total protein extracts prepared from either cells or TA muscles samples. TA muscles were homogenized with TissueLyserII (Qiagen) following manufacturer's instructions. These primary antibodies were used: Cripto (1:500, R&D System), eMyHC (1:100, Developmental Studies Hybridoma Bank), Gapdh (1:40.000, Abcam) and β-Actin (1:5000, Sigma-Aldrich).

### Isolation of mouse satellite cells and in vitro differentiation

*Tg:Pax7CreER^T2^::Cripto* and *Tg:Cripto* new-born mice were injected i.p. with tamoxifen (10 μM, 5 μl) every day, from day 1 to day 5 after birth (P1–P5). Preparation of MPCs from new-born mice (P7) was performed as previously described (Rando and Blau, 1994). Briefly the hindlimb and forelimb were removed and separated from the bones. Muscles were mechanically minced with surgical scissors and dissociated by enzymatic digestion [600 U/ml Collagenase Type IV (Gibco) and 1.8 U/ml Dispase II (Roche) in DMEM (Gibco)] at 37°C for 2 h in the shaker. Digested muscles were spinned at 500 rpm for 5′ and satellite cells were spinned down from supernatant (1500 rpm for 10′). Cells were plated on gelatin-coated dishes in proliferation medium [1% Pen/Strep (EuroClone), 1% Glutamin (Euroclone), 20% FBS (Euroclone), 2% UltraSerum (LifeScience) in DMEM (Gibco),] and myoblast population was enriched by several pre-plating steps (Richler and Yaffe, 1970). For Pax7/MyoD cell counting, MPCs were cytospinned at a density of 50.000 cells/spot after 24 h in proliferating medium. For myogenic differentiation, myoblasts were plated in 48 wells at a density of 2 × 10^4^ cells/cm^2^ in proliferation medium. After twenty-four hours, proliferation medium was replaced with differentiation medium for 24–72 h [DMEM (Gibco) containing 2% (v/v) horse serum (Gibco) and 1% pen/strep (EuroClone)] and the fusion index was determined as the ratio of the number of nuclei in myotubes (MF20^+^ cells with *n* ≥ 2 nuclei) on the total nuclei (MF20^+^ cells).

### Immunofluorescence analysis

Cells were fixed for 10′ with 4% (wt/vol) paraformaldehyde (PFA) in Phosphate Buffer Saline (PBS) and blocked with 1% bovin serum albumin (BSA) in PBS. Primary antibodies used were: Cripto (Parisi et al., 2003), MF20 (1:50, Developmental Studies Hybridoma Bank), Pax7 (1:10, Developmental Studies Hybridoma Bank) and MyoD (1:50, SantaCruz). Appropriate fluorophore-conjugated secondary antibodies, Alexa Fluor 594 or 488 (1:600, Molecular Probes), biotin-conjugated goat anti-mouse (Jackson), and Cy3- (Jackson) or 488- (Molecular Probes) conjugated streptavidin were used for the visualization using DMI600 or DM600 (Leica) fluorescence microscopes.

Muscles were freshly frozen and cut in cryostat sections. Slides were fixed in 4% (wt/vol) PFA and processed as previously described (Nicolas et al., 2005). Briefly, muscle sections were permeabilized in ice-cold methanol for 6′ at −20°C, and boiled 15 min in 10 mM sodium citrate pH 6. Unbinding sites were blocked with 4% IgG-free BSA (Jackson) in PBS for 2–3 h at RT followed by a second blocking step with AffiniPure Fab Fragment goat anti-mouse IgG (Jackson). Primary antibodies were as follows: eMyHC (1:50, F1.652-s, Developmental Studies Hybridoma Bank), Pax7 (1:10, Developmental Studies Hybridoma Bank) and MyoD (1:50, SantaCruz) Appropriate fluorophore-conjugated secondary antibodies, Alexa Fluor 488 or 594 (1:600, Molecular Probes), biotin-conjugated goat anti-mouse (Jackson) and Cy3- (Jackson) or 488- (Molecular Probes) conjugated streptavidin were used for visualization. Nuclei were counterstained with DAPI (1:100 in PBS, VinciBiochem) and FluoSave Reagent (Calbiochem) was used for mounting. Labeling was visualized by epifluorescent illumination using a DM600 microscope (Leica), and images were acquired on a DFC360-FX camera (Leica).

### ELISA-based assay

ELISA–based assay was performed as previously described (Guardiola et al., 2012). Briefly ninety-six well plates were coated with 0.5 μg/ml of sheep anti-mouse Cripto Ab (R&D System, AF1538) in PBS overnight (O.N.) at 4°C. Unbinding sites were blocked with PBS-BSA 1% for 2 h at RT. After washing, protein extracts (300 μg) were added and plates were incubated O.N. at 4°C. The plates were incubated with 1 μg/ml of mouse Cripto biotinylated Ab (R&D System, BAF 1538) in PBET (0.1% BSA, 5 mM EDTA, 0.004% Tween-20 in PBS 1X pH 7,5) for 1 h at 37°C, and then for 1 h at RT. Finally, the plates were incubated for 1 h at RT with avidin/streptavidin complex conjugated with horse-radish peroxidase (Vectastain elite ABC kit, Vector Laboratories). The signals were visualized with *o*-phenylenediamineperoxidase substrate (OPD, Sigma-Aldrich) and the relative absorbance was read at 490 nm on a Benchmark microplate reader (Bio-Rad Laboratories).

### RNA preparation and qRT-PCR

TA muscles were homogenized with TissueLyserII (Qiagen) in TriReagent (Life Technologies) and total RNA was isolated according to the manufacturer's instructions. One microgram of total RNA was utilized for cDNA synthesis using SuperScript II Reverse Transcriptase and random hexamers (Qiagen). qRT-PCR was performed using SYBR Green PCR master mix (EuroClone). Primers are listed in Table S2.

### Statistical analysis

All values are expressed as mean ± Standard Error of the Mean (SEM). To determine significance between two groups, comparisons were made using unpaired Student *t*-tests. ^*^P ≤ 0.05, ^**^P ≤ 0.005, and ^***^P ≤ 0.0005 were considered statistically significant.

## Author contributions

CP, design of the work, data acquisition and analysis, data interpretation. SI, data acquisition and analysis, data interpretation. GA, LZ, FI, data acquisition and analysis. OG, conception and design of the work, data analysis, data interpretation and draft of the article. GM, conception and design of the work, data interpretation, writing and revising of the article. All the authors have read and approved the final manuscript.

### Conflict of interest statement

The authors declare that the research was conducted in the absence of any commercial or financial relationships that could be construed as a potential conflict of interest.
