# Supplementary material for: Conditional Cripto overexpression in satellite cells promotes myogenic commitment and enhances early regeneration
Source: Front Cell Dev Biol. 2015 May 21;3:31. doi: 10.3389/fcell.2015.00031 (PMC4439575; doi:10.3389/fcell.2015.00031)
Supplement: Supplementary file 1 [file DataSheet1.DOCX]

**Supplementary Material**

**Conditional Cripto overexpression in satellite cells promotes myogenic commitment and enhances early regeneration**

Carolina Prezioso^1^, Salvatore Iaconis^1^, Gennaro Andolfi^1^, Lorena Zentilin^2^, Francescopaolo Iavarone^1^, Ombretta Guardiola^1*^ and Gabriella Minchiotti^1*^

^1^ Stem Cell Fate Laboratory, Institute of Genetics and Biophysics “A. Buzzati-Traverso”, CNR, Naples, Italy

^2^ Molecular Medicine Laboratory, International Centre for Genetic Engineering and Biotechnology, Trieste, Italy

**Supplementary Figures and Tables**

**Figure S1.** **(A)** Representative pictures of direct fluorescence of HEK-293T cells at 48 hours after transfection with pDsRed^loxP/loxP^Cripto and pCMV-Cre. Scale bars=100μm. **(B)** Representative pictures of immunofluorescence with anti-Cripto (red) antibody on HEK-293T cells at 48 hours after transfection with pDsRed^loxP/loxP^Cripto and pCMV-Cre. Scale bars=100μm.

**Figure S2. (A)** PCR on muscle genomic DNA showing tamoxifen-induced recombination only in the muscle of *Tg:Pax7CT2::Cripto^(B)^*. Primers c-c’ (See Figure 1C) amplified a 1995-bp fragment of the transgenic allele and a 838-bp fragment of the recombining allele. The recombining allele is detected in the muscle (M; lane 1) but not in the liver (L; lane 3) of *Tg:Pax7CT2::Cripto^(B)^* mice, and is not detected in the muscle (M; lanes 2) and in the liver (L; lane 4) of *Tg:Cripto^(A)^* control littermates. **(B)** ELISA assay of Cripto protein levels in TA muscles after CTX injection expressed as pg/mg of muscle tissue at the indicated time points. **(C)** Representative pictures of embryonic Myosin Heavy Chain (eMyHC) immunofluorescence on TA sections from *Tg:Pax7CT2::Cripto^(B)^* mice and *Tg:Cripto^(B)^* control littermates at day 8. Scale bars=100μm. **(D-F)** qRT-PCR analysis of neonatal Myosin Heavy Chain (nMyHC) **(D)** Myogenin (Myog) **(E)** and Myostatin (Mstn) **(F)** in TA muscles from *Tg:Pax7CT2::Cripto^(B)^* and the *Tg:Cripto^(B)^* control littermates at day 8 after CTX injection. Values are mean ± SEM, 3 mice/group. *P≤ 0.05.

**Figure S3. (A)** Representative H&E staining of TA muscle sections from *Tg:Pax7CT2::Cripto^(B)^* and *Tg:Cripto^(B)^* mice at the indicated time points. Scale bars=50µm. **(B)** Morphological analysis on muscle sections at day 8 after injury showing the number of myofibers containing more than one central nuclei/area in each group. **(C)** Average of centrally nucleated myofibers size in TA muscle sections at day 8 after injury of *Tg:Pax7CT2::Cripto^(B)^* and *Tg:Cripto^(B)^* control littermates. **(D)** Myofiber Cross Sectional Area distribution at 15 days after CTX injection in *Tg:Pax7CT2::Cripto^(B)^* and *Tg:Cripto^(B)^* mice. **(E)** Average of centrally nucleated myofibers size values in TA muscle sections at day 15 after injury of *Tg:Pax7CT2::Cripto^(B)^* mice and *Tg:Cripto^(B)^* control littermates. (**F**) Myofiber Cross Sectional Area distribution at 30 days after CTX injection in *Tg:Pax7CT2::Cripto^(B)^* and *Tg:Cripto^(B)^* mice. Values are mean ± SEM, 5 mice/ group. *P≤ 0.05.

**Figure S4. (A)** Representative pictures of double immunofluorescence with antibodies anti-Pax7 and anti-MyoD in postnatal MPCs isolated from newborn (P7) *Tg:Pax7CT2::Cripto^(A)^* mice and their *Tg:Cripto^(A)^* control littermates. Nuclei were counterstained with DAPI. Arrowheads indicate Pax7^-^/MyoD^+^ (white) cells. Scale bars= 100µm. **(B)** Effect of Cripto overexpression on Pax7^-^/MyoD^+^ cell population *in vitro*. Pax7^-^/MyoD^+^ cells were calculated as percentage of total cell counts and reported (9.92 ± 1.88% for *Tg:Pax7CT2::Cripto^(A)^* vs 3 ± 1.62% for control *Tg:Cripto^(A)^*). Values are mean ± SEM of 3 indipendent experiments. *P≤0.05.

**Table S1**

| **Primer** | **Forward 5'-3'** | **Primer** | **Reverse 5'-3'** |
| --- | --- | --- | --- |
| a | CGCAACTGTGAACATGATGT | a' | CACAGCCAGGTAGAAAGGTC |
| b | GAACGTCATCACCGAGTTCA | b' | GGGTGCTTCACGTACACCTT |
| c | GAGCCTCTGCTAACCATGTTC | c' | CACAGCCAGGTAGAAAGGTC |
| DsRedCriptofor | GGGTGCTTCACGTACACCTT | DsRedCripto rev | GAACGTCATCACCGAGTTCA |
| Pax7Cre  for | CCACACCTCCCCCTGAACCTGAAAC | Pax7Cre  rev | GAATTCCCCGGGGAGTCGCATCCTG |

**Table S2**

| **Primer** | **Forward 5'-3' sequence** | **Reverse 5'-3' sequence** | **Gene ID** |
| --- | --- | --- | --- |
| *GAPDH* | TGCACCACCAACTGCTTAGC | TCTTCTGGGTGGCAGTGATG | 14433 |
| *nMyHC* | GAACTTGAAGGAGAGGTCGA | GATTTACCAATGCCTTGTAG | 111671 |
| *Myog* | CTACAGGCCTTGCTCAGCTC | CACGATGGACGTAAGGGAGT | 17928 |
| *Mstn* | TGTAACCTTCCCAGGACCAG | TCTTTTGGGTGCGATAATCC | 17700 |
