# Supplementary figures and images for: Conditional Cripto overexpression in satellite cells promotes myogenic commitment and enhances early regeneration
Source: Front Cell Dev Biol. 2015 May 21;3:31. doi: 10.3389/fcell.2015.00031 (PMC4439575; doi:10.3389/fcell.2015.00031)

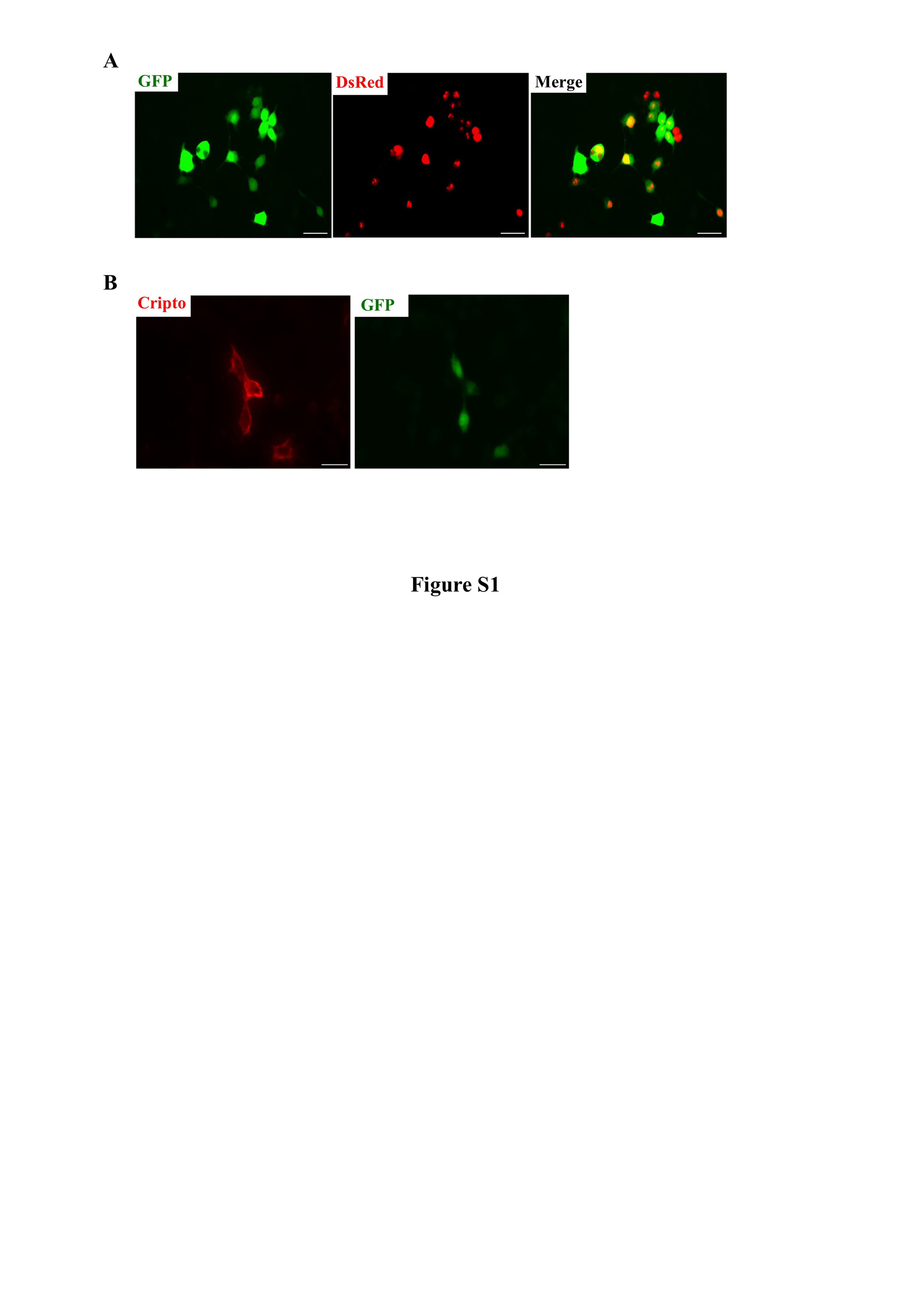

Supplement: Supplementary file 2 [file Image1.JPEG]

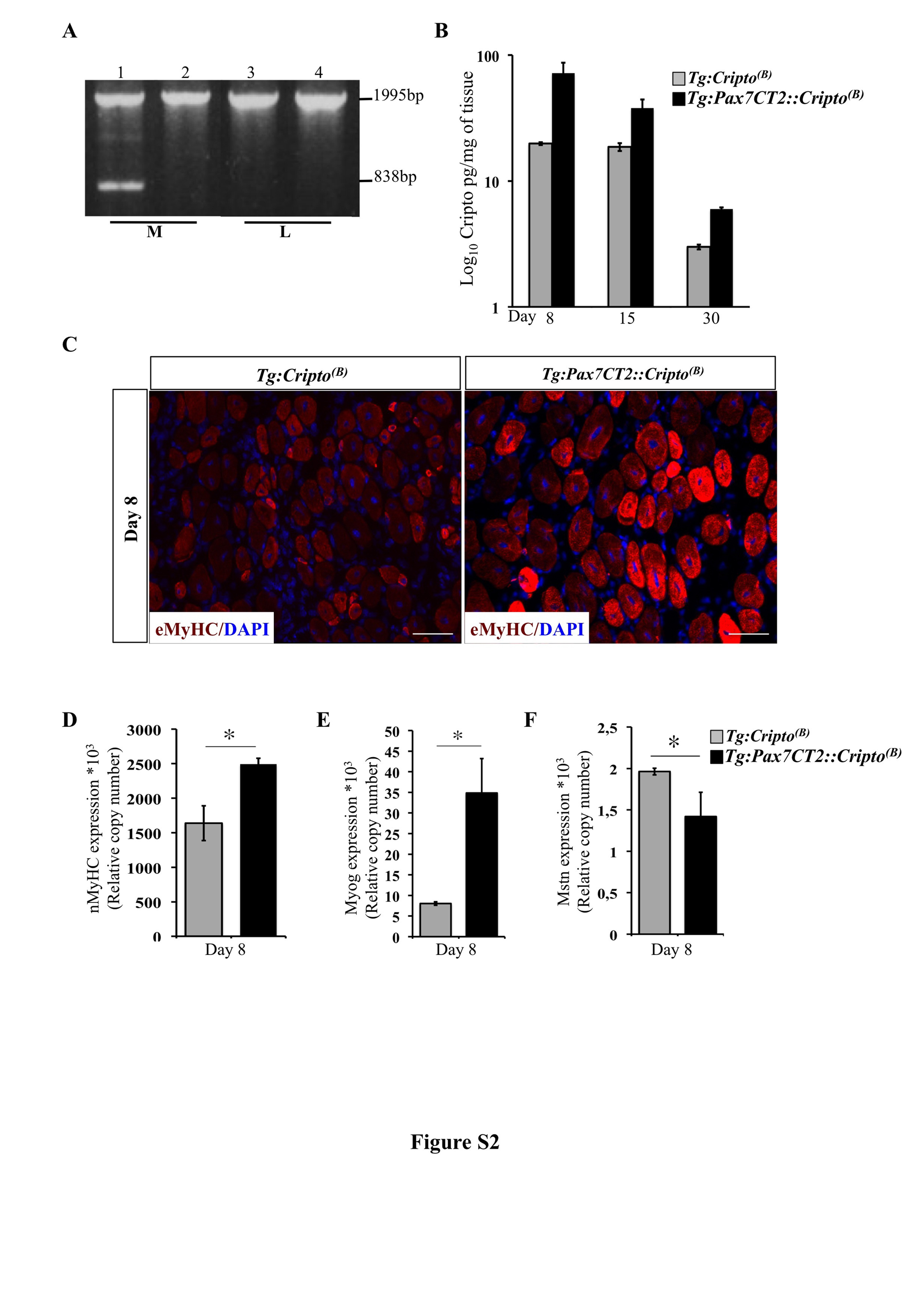

Supplement: Supplementary file 3 [file Image2.JPEG]

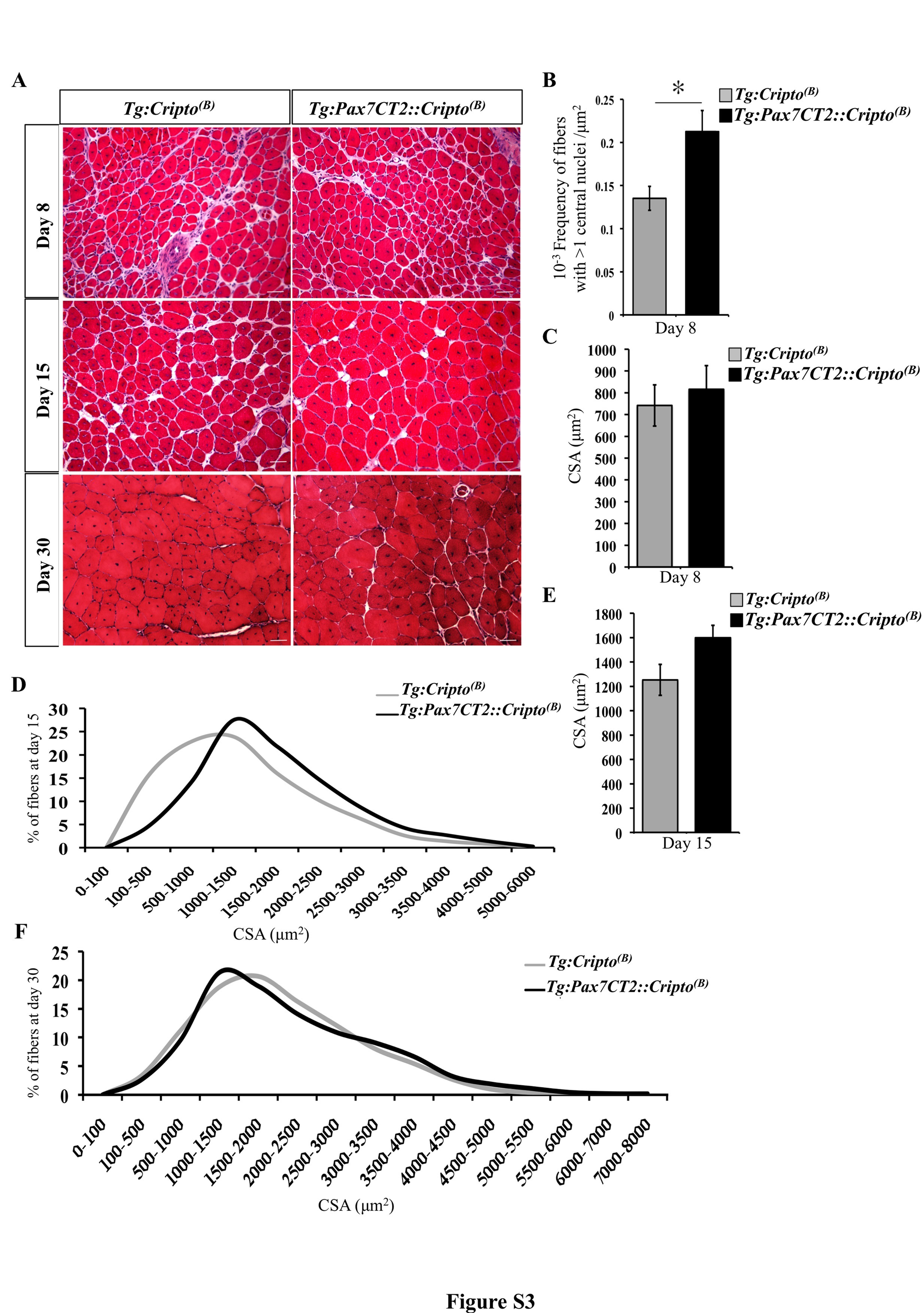

Supplement: Supplementary file 4 [file Image3.JPEG]

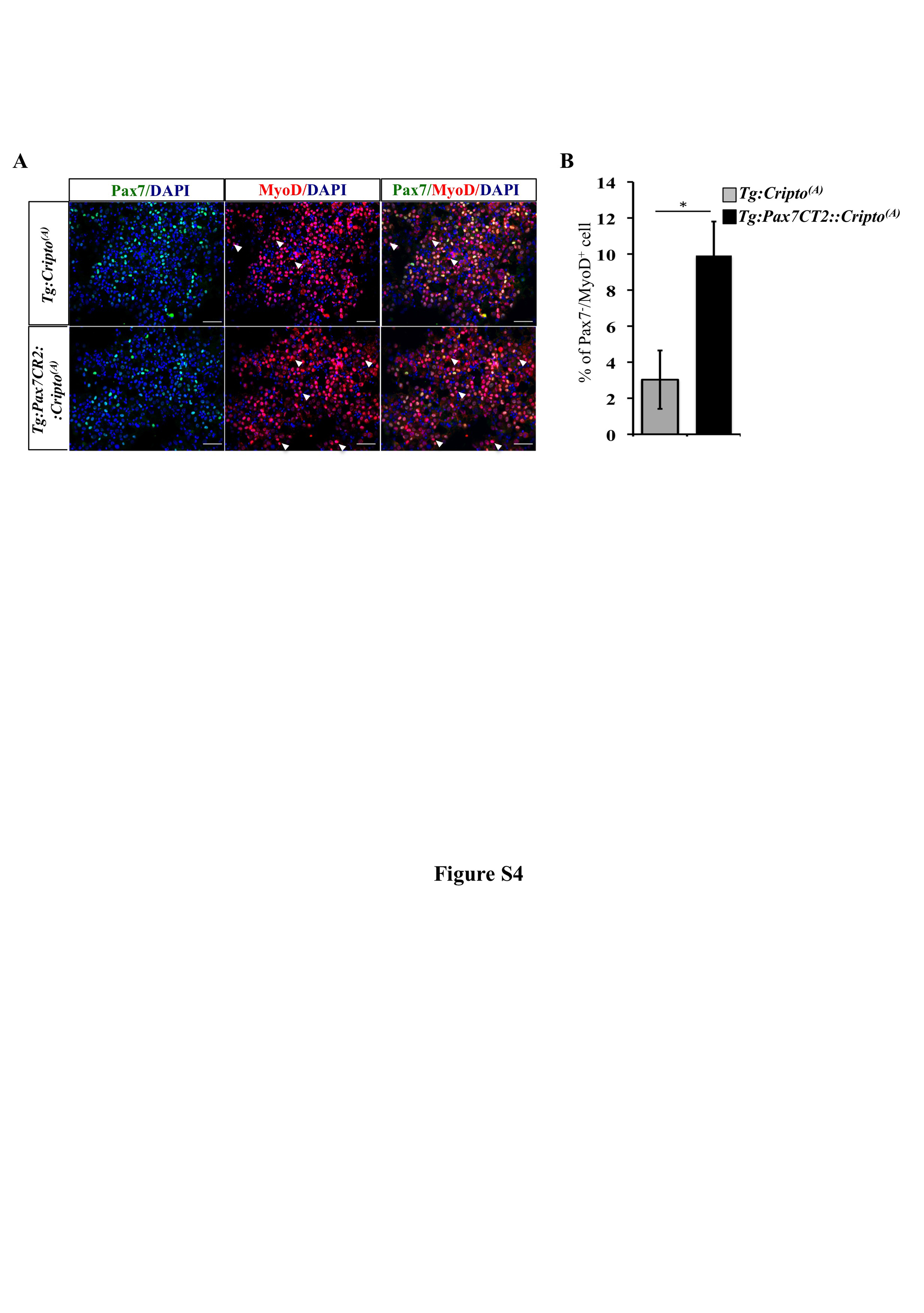

Supplement: Supplementary file 5 [file Image4.JPEG]
